# Flexible Three-Dimensional Stress Sensor for Embedded Monitoring of Solid Rocket Propellant

**DOI:** 10.3390/mi17010057

**Published:** 2025-12-30

**Authors:** Yaoguang Shi, Xiaozhou Lü, Kai Ren, Wensong Zhu

**Affiliations:** School of Aerospace Science and Technology, Xidian University, Xi’an 710071, China

**Keywords:** flexible three-dimensional stress sensor, solid rocket motor, health monitoring, liquid metal

## Abstract

Solid rocket motors (SRMs) play a pivotal role in space exploration owing to their reliability and high thrust-to-weight ratios. SRM propellant health monitoring is in critical demand owing to the complex operational scenarios throughout the entire life cycle of SRMs. To achieve in situ detection of three-dimensional stress, this study introduces a novel flexible three-dimensional stress sensor (FSS). First, a liquid metal pressure-sensing element with a variable cross-section was designed and numerically modeled. The performance of the FSS under different loading conditions was analyzed using finite element modeling. The sensing element prototype was fabricated using mold casting and liquid metal injection methods. The fabricated sensing-element prototype with an area ratio of 1:5 exhibited a sensitivity coefficient of 1.5%/kPa at a pressure of 300 kPa, a maximum hysteresis error of 3.98%, and a stability error of 0.17%. Finally, the FSS was developed by integrating multiple pressure-sensing elements and encapsulating the force-concentrating layers. The fabricated FSS prototype was characterized using simulated propellant experiments. Via comparison with the simulation results, the FSS was found to detect multiaxial stress differences when embedded within a propellant.

## 1. Introduction

Solid rocket motors (SRM) are widely used in the propulsion systems of aerospace vehicles and offer numerous advantages, such as a simple structure and long storage life [1]. The propellant, which is the core component of SRMs, is typically composed of viscoelastic polymeric materials [2,3]. Throughout the full life cycle of a SRM, the propellant faces various complex conditions. For example, prolonged storage may cause deformation of the propellant under gravity, and fluctuations in ambient temperature and vibrations during transportation may lead to internal cracks or interface debonding of the propellant, affecting the reliability and safety of the SRM. Therefore, propellant health monitoring is required [4].

Currently, the health monitoring of SRM propellants mainly includes destructive testing, nondestructive testing, modeling analysis, and the use of embedded sensors [5]. Destructive testing includes visual inspection of the components of the SRM, which is the most basic form of inspection and involves looking for signs of defects on the surface of the SRM, such as cracks, bulges, and discoloration [6]. Nevertheless, destructive testing cannot guarantee the detection of every potential problem or failure scenario because it is performed on a limited number of samples considered representative of a specific SRM population. Non-destructive testing techniques are non-in situ testing methods, including ultrasonic holography [7,8], X-rays [9,10,11], infrared rays [12], and laser holography (speckle) non-destructive testing [13]. For instance, Liu et al. developed a high-resolution synchrotron X-ray tomography to investigate the damage mechanism of propellants [11]. Liu et al. proposed a multi-scale image enhancement algorithm for de-bonding defects in solid propellant rocket motors, and the vast majority of noise can be eliminated using the developed method [12]. However, non-destructive testing techniques also have limitations in terms of inspection depth, sensitivity to certain defect types, and limited access to certain areas of SRMs. The modeling analysis method establishes mathematical or numerical models of the propellant and evaluates its health status through computational analysis [14,15,16]. For instance, Lei et al. developed a finite element model to show the correlation between propellant failure and propellant–liner debonding, which can predict both the strain field at the crack tip and the stress–strain response during interface debonding at an ultra-low temperature of −60 °C. However, modeling analysis is difficult to achieve in situ and for real-time monitoring of the internal state of SRM propellants. The embedded sensor monitoring method mainly uses interface sensors [17], fiber-optic sensors [18,19,20], and flexible strain sensors [21,22,23] to realize in situ internal stress or strain detection of propellants. The above real-time monitoring method can detect interface stress or debonding and strain of internal cavities; however, three-dimensional stress gradient changes inside the propellant have not yet been achieved. Numerous flexible three-dimensional force sensors have been developed for tactile sensing, intelligent robotics, wearable healthcare monitoring, and human-machine interaction [24,25,26,27]. However, these three-dimensional force sensors decouple the normal and shear forces when mounted on the human body or dexterous hand, rather than the embedded three-dimensional stress detection in the spatial contexts of the SRM propellant.

In response to the above issues, this study proposes an embedded flexible three-dimensional stress sensor (FSS) for SRM propellants, capable of real-time monitoring of multiaxial stress differences within propellants. First, we conducted a structural design of the variable cross-sectional liquid metal pressure-sensing element and the FSS. The sensitivity improvement mechanism of the pressure-sensing element and stress difference of the FSS in the simulated propellant were investigated using finite element simulations. Subsequently, prototypes of the pressure-sensing element and FSS were fabricated, and the stress detection ability of the FSS was verified using simulated propellant grains.

## 2. Principle

### 2.1. Sensing Mechanism

**Figure 1:**
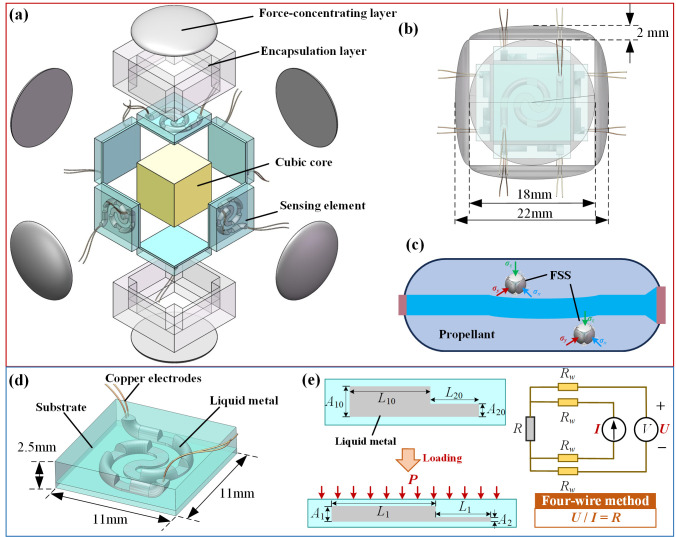
(**a**) Schematic diagram, (**b**) structural dimensions, and (**c**) working principle of the FSS embedded in the SRM propellant, (**d**) schematic diagram, and (**e**) working mechanism of the liquid metal pressure-sensing element with a variable cross-section.

The structural design of the proposed FSS is shown in Figure 1a, which consists of a cubic core, six pressure-sensing elements, two flexible encapsulation layers, and six force-concentrating layers. A cubic core with properties similar to those of the propellant was located at the center. Pressure-sensing elements with consistent structures were attached to the outer surfaces of the cubic core, which could detect stress differences in three-dimensional directions. Flexible encapsulation layers were integrated on the outside of the pressure-sensing elements to facilitate their integration. The force-concentrating layers corresponding to each pressure-sensing element were situated on the outermost layer, which was utilized to enhance the stress concentration capability of the sensor. The overall dimensions of the FSS were 22 mm × 22 mm × 22 mm, and the force-concentrating layer was 18 mm × 18 mm × 2 mm, as shown in Figure 1b. Owing to the similar modulus of the propellant and the symmetrical structure, the proposed FSS can sensitively detect three-dimensional stress differences without affecting the mechanical properties of the propellant. As shown in Figure 1c, the difference in stress between the two coaxially paired sensing elements of the FSS embedded in the SRM propellant enables the detection of the uniaxial stress difference during prolonged storage in the SRM. Therefore, the six sensing elements can detect three-dimensional stress differences. To improve the sensitivity, the pressure-sensing elements were designed with liquid metal as a piezoresistive material encapsulated in a variable cross-sectional channel, as illustrated in Figure 1d. The dimensions of the variable cross-section pressure-sensing element were designed to be 11 mm × 11 mm × 2.5 mm. The resistance network changes in the variable cross-section liquid metal pressure-sensing elements are illustrated in Figure 1e. When normal stress is applied to the sensing element, the cross-sectional area of the elastomer channel decreases, resulting in a narrowing of the conductive network of the liquid metal and an increase in resistance. When a microchannel with a small cross-sectional area is subjected to a large pressure, it approaches blockage and causes an obvious resistance variation. Therefore, the variable cross-section liquid metal pressure sensing element can enhance the sensitivity of the FSS. Because the liquid metal has an ultralow baseline resistance, the copper electrodes were set as the four-wire method in the pressure-sensing element, where the voltage drop in the test leads was eliminated when the voltage was measured.

### 2.2. Mathematical Modeling of the Pressure-Sensing Element with a Variable Cross-Section

The liquid metal sensing element with a variable cross-section was simplified into two segments, each with different lengths and cross-sectional areas. The initial electrical resistance (*R*_0_) of the liquid metal-sensing element can be defined as follows:

(1) $$R_{0}=R_{10}+R_{20}=\rho \left(\frac{L_{10}}{A_{10}}+\frac{L_{20}}{A_{20}}\right)$$

where *R*_10_ and *R*_20_ are the initial electrical resistances of the wide and narrow cross-sections, respectively; *ρ* is the electrical resistivity of the liquid metal; *L*_10_ and *L*_20_ are the initial lengths of the wide and narrow cross-sections, respectively; and *A*_10_ and *A*_20_ are the initial areas of the wide and narrow cross-sections, respectively.

As shown in Figure 1e, when pressure (*P*) is applied to the sensing element, the resistance of the liquid metal varies owing to the deformed channel. The strain along the pressure for each segment is defined as *ε_yi_*, which is the strain in the height of each channel and perpendicular to the direction of the elongated channel. The strain (*ε_xi_*) along each channel can be defined as follows:

(2) $$\varepsilon_{xi}=\frac{\varepsilon_{yi}}{-\mu }=\frac{PA_{s}}{\mu EA_{i0}}$$

where *A_s_* is the applied area of the sensing element, *μ* is the Poisson’s ratio, and *E_i_* is the Young’s modulus of the channel. Because the wide channel has a thinner encapsulation, its modulus is smaller than that of the narrow channel. Then, the electrical resistance (*R_i_*) of each segment of the liquid metal-sensing element under pressure can be calculated as follows:

(3) $$R_{i}=\rho \left(\frac{L_{i0}(1+\varepsilon_{xi})}{A_{i0}{\left(1-\mu \varepsilon_{xi}\right)}^{2}}\right)$$

where *L_i_*_0_ and *A_i_*_0_ are the initial length and area of the different segment. The cross-sectional area and length ratios are represented as

(4) $$n=\frac{A_{10}}{A_{20}}$$

(5) $$m=\frac{L_{10}}{L_{20}}$$

Thus, the relative resistance variation can be calculated as

(6) $$\frac{R-R_{0}}{R_{0}}=\sum_{i=1}^{2}\frac{R_{i0}}{R_{0}}\frac{R_{i}-R_{i0}}{R_{i0}}$$

where *R* is the electrical resistance of the liquid metal. By substituting Equation (2) to Equation (6), the relative resistance variation can be performed as a second-order Taylor expansion as follows:

(7) $$\frac{R-R_{0}}{R_{0}}\approx \frac{1}{1+\frac{m}{n}}\left(\left(2\mu +1\right)\frac{PA_{s}}{\mu EA_{10}}+\left(2\mu +3\mu^{2}\right){\left(\frac{PA_{s}}{\mu EA_{10}}\right)}^{2}\right)+\frac{m/n}{1+\frac{m}{n}}\left(\left(2\mu +1\right)\frac{PA_{s}}{\mu E{nA}_{10}}+\left(2\mu +3\mu^{2}\right){\left(\frac{PA_{s}}{\mu EnA_{10}}\right)}^{2}\right)$$

Therefore, the relative resistance variation has a nonlinear relationship with the applied pressure. In addition, the cross-section ratio significantly improves sensitivity.

## 3. Finite Element Analysis

### 3.1. Modeling of Pressure Sensing Element

**Figure 2:**
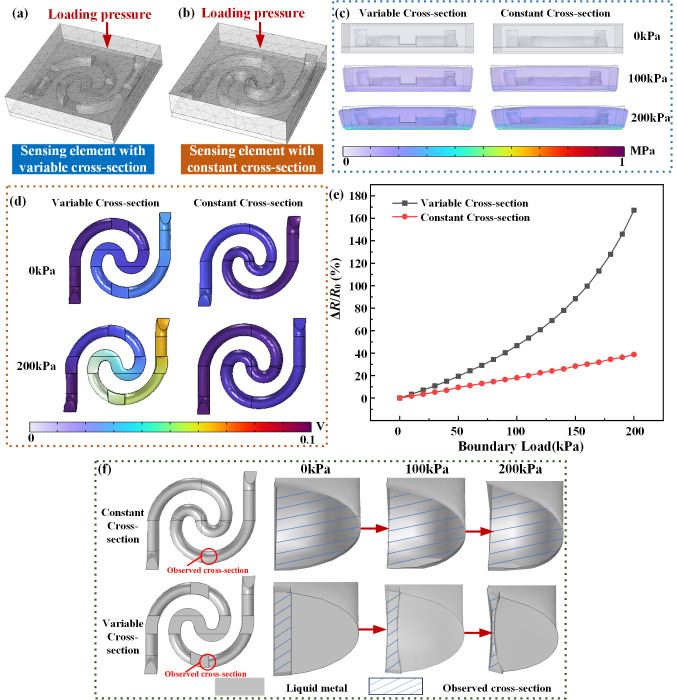
3D model of liquid metal pressure-sensing element with (**a**) variable cross-sectional channel and (**b**) constant cross-sectional channel structure; (**c**) stress distribution, (**d**) electric potential distribution, and (**e**) resistance variation of different sensing elements under pressure; (**f**) deformation comparison of cross-section in different sensing elements under pressure.

The proposed FSS is intended to be fabricated using a silicone rubber elastomer with an elastic modulus similar to that of propellant. To study the impact of variable cross-sectional channel structures on the sensitivity of pressure-sensing elements, a three-dimensional model was established using the commercial finite element analysis software COMSOL Multiphysics 6.2. The flexible encapsulation material of the pressure-sensing element was set with a Young’s modulus of 1.48 MPa and a Poisson’s ratio of 0.49. The electrical conductivity, density, and dynamic viscosity of the liquid metal were set to 3.46 × 10^6^ S/m, 6.44 g/cm^3^, and 0.0024 Pa·s, respectively. To investigate the advantages of the variable cross-section sensing element, another model of a pressure-sensing element with a constant cross-section was established, as shown in Figure 2a,b, respectively. The boundary conditions in the simulation were as follows: the sensing element in the solid mechanics module had an externally supported bottom surface set as a fixed constraint, and the top of the sensing element was set as the boundary load surface with an applied pressure increasing from 0 to 200 kPa in increments of 10 kPa. As shown in Figure 2c, both sensing elements exhibited significant deformation of the sensing element under a pressure of 200 kPa. The maximum stress of both sensing elements was similar, and the relative area change rate of the narrow section in the variable cross-section channel increased significantly.

To investigate the resistance variation of the different sensing elements, a constant current of 1A was applied to both ends of the liquid metal, and the resistance changes were calculated based on the output voltage values. The voltage distributions of the sensor under different pressures are shown in Figure 2d. The initial voltage of the liquid metal pressure-sensing element with a variable cross-section was higher, and as the pressure increased, the voltage variation was greater. The relative resistance changes of the liquid metal pressure-sensing elements under different pressures are shown in Figure 2e. As the loading pressure increased, the relative resistance variation in the liquid metal pressure-sensing element with a variable cross-section increased fourfold under 200 kPa. The results confirm that the variable cross-sectional channel design enhances the sensitivity of the liquid metal pressure-sensing element. As shown in Figure 2f in the revised manuscript, the cross-section of the liquid metal channel in the pressure-sensing element varied according to the applied pressure. Owing to the near incompressibility of the flexible encapsulation material, the variation mainly occurred in the narrow channel.

### 3.2. Modeling of FSS Embedded in Propellant

#### 3.2.1. Uniaxial Normal Loading Simulation

**Figure 3:**
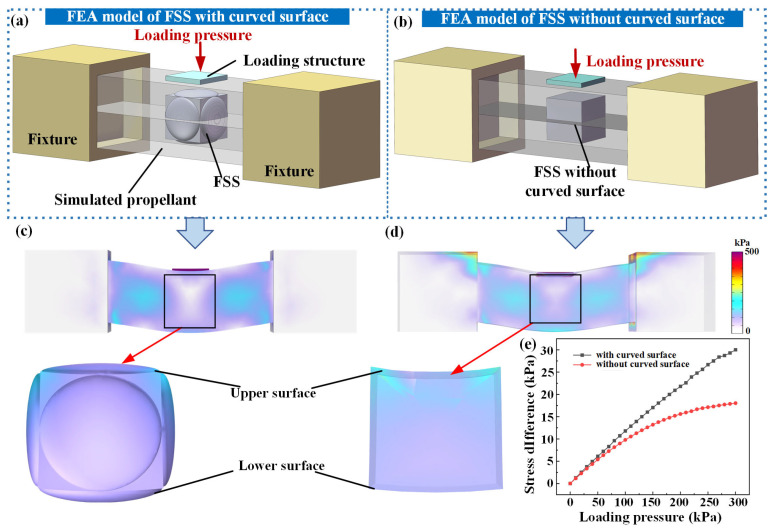
FSS (**a**) with and (**b**) without a force-concentrating layer embedded in the simulated propellant; stress distribution of the FSS under loading (**c**) with and (**d**) without a force-concentrating layer; (**e**) stress differences of the different FSS.

To investigate the deformation performance of the FSS embedded within the propellant during prolonged storage, different three-dimensional models of the FSS with and without a force-concentrating layer were established. Figure 3a,b show the three-dimensional structural models of the propellant-embedded FSS with and without a force-concentrating layer, respectively. In the simulation, the Young’s modulus of the propellant was set to 1.53 MPa with a Poisson’s ratio of 0.49, whereas the rigid loading structure exhibited a Young’s modulus of 346 MPa and Poisson’s ratio of 0.35. To impose mechanical loading, uniaxial normal pressure from 0 to 300 kPa in increments of 10 kPa was applied to the top surface of the loading structure, enabling a systematic analysis of the stress distribution under controlled loading conditions. Figure 3c,d show the deformation distributions of the different FSS in the simulated-propellant module. The sensor with the force-concentrating layer experienced significantly greater deformation, which improved the resistance variation in the sensing elements. The stresses on the upper and lower surfaces and the stress difference between the upper and lower surfaces of the FSS with and without a force-concentrating layer are shown in Figure 3e. As the loading pressure increased, the force-concentrating layer enhanced the stress concentration, and the stress detected by the upper surface and stress difference were significant, which helped the sensing element capture minute changes in the internal three-dimensional stress of the propellant.

#### 3.2.2. Multiaxial Loading Simulation

**Figure 4:**
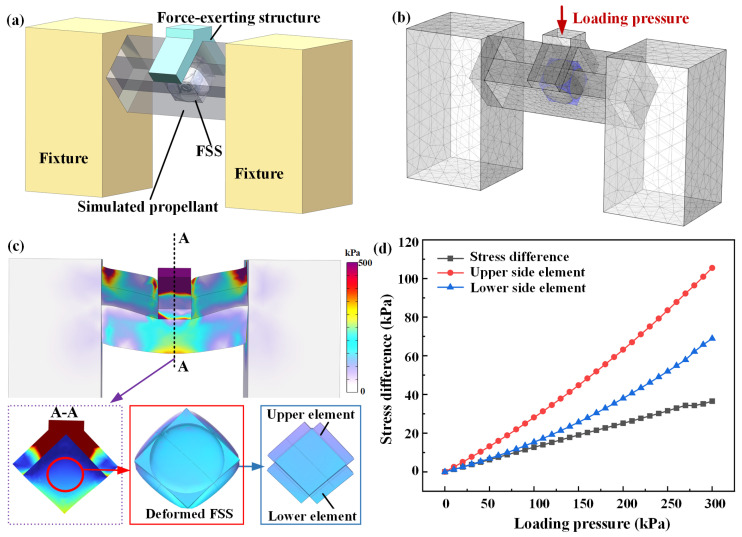
(**a**) 3D model of the FSS embedded in the simulated propellant under composite direction loading, (**b**) meshed grids, (**c**) stress distribution, and (**d**) stress difference of the FSS under multiaxial loading.

To investigate the multiaxial stress detection ability of the embedded FSS in an SRM propellant during prolonged storage, a force-exerting structure was added outside the simulated propellant, which can load pressure in two directions within the propellant, thereby more realistically simulating a complex three-dimensional stress environment, as shown in Figure 4a,b. A loading pressure ranging from 0 to 300 kPa was applied to the top of the force-exerting structure in increments of 10 kPa. Simultaneously, all the surfaces of the fixtures were set with fixed constraints. As shown in Figure 4c, the deformation of the upper inclined surface of the FSS was significantly greater than that of the lower inclined surface, resulting in the upper sensing elements experiencing a greater force than the lower ones. Figure 4d illustrates the stress of the upper and lower sensing elements and the variation in the stress difference for different loads. As the loading pressure increased, the change in stress for the upper-side sensing element was higher than that for the lower-side sensing element; thus, the stress difference gradually increased. At a load of 300 kPa, a stress difference of approximately 75 kPa was obtained, which reflected the direction of the loading applied to the propellant.

## 4. Fabrication Procedure

### 4.1. Preparation of Liquid Metal Pressure Sensing Element

**Figure 5:**
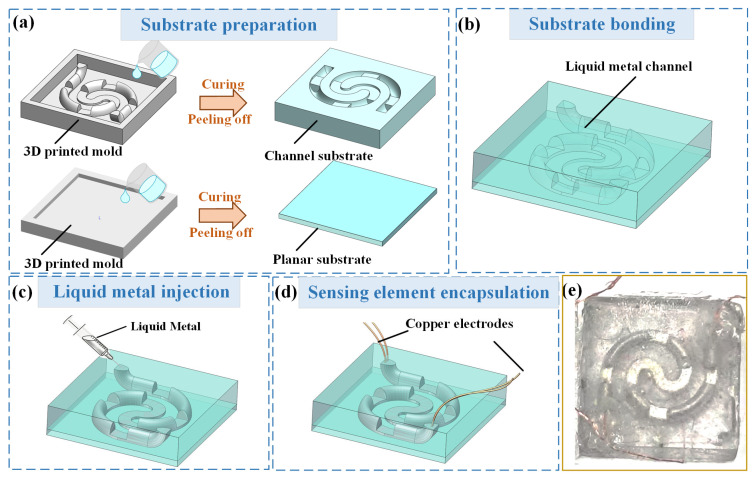
Fabrication procedure of the liquid metal pressure-sensing element with a variable cross-section: (**a**) substrate preparation, (**b**) substrate bonding, (**c**) liquid metal injection, (**d**) sensing element encapsulation, and (**e**) photograph of the pressure-sensing element prototype.

A liquid metal pressure-sensing element with a variable cross-section was fabricated using mold casting and injection-filling processes. The detailed preparation process for the sensing element was as follows:

(1) Substrate preparation: The PDMS (Sylgard 184 silicone, Dow Inc., Midland, MI, USA) prepolymer and curing agent were mixed in a weight ratio of 10:1 in a beaker using magnetic stirring, and the mixture was placed in a vacuum oven for degassing. As shown in Figure 5a, 3D-printed molds with positive structures corresponding to the channels and a planar groove were pretreated by spraying a release agent on them. The PDMS prepolymer was poured into the 3D-printed molds and cured at 60 °C for 3 h. Once fully cured, the channel and planar substrates were peeled off from the molds.

(2) Substrate bonding: During the manufacturing process of the liquid metal sensing element, the silicone sealant can be spread evenly owing to its high adhesion, which is significant for element sealing and can effectively prevent liquid metal leakage, thus improving the sealing and reliability of the device. As shown in Figure 5b, a layer of silicone sealant was evenly coated on the surface of the peeled substrates, and the microchannel and planar substrates were bonded at RT.

(3) Liquid metal injection: The EGaIn alloy (68.5 wt% gallium, 21.5 wt% indium, and 10 wt% tin) provided by Santech Materials Co., Ltd., Changsha, China was used as the liquid metal-sensitive material. As shown in Figure 5c, the liquid metal was injected into the microchannel using a syringe. Four copper wires were inserted into both sides of the liquid metal to form four-wire method electrodes, which facilitated the measurement of the low resistance of the liquid metal.

(4) Sensor element encapsulation: As shown in Figure 5d, the PDMS prepolymer was added to the electrodes and cured for 4 h. The fabricated prototype of the liquid metal pressure-sensing element with a variable cross-section is shown in Figure 5e.

### 4.2. Integration of FSS

**Figure 6:**
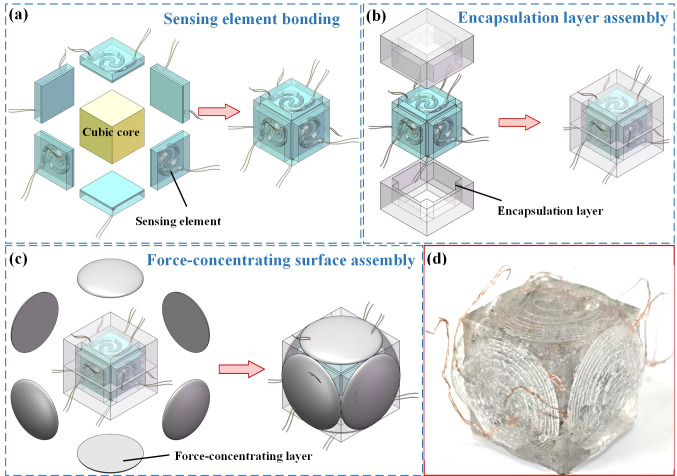
Integration procedure of the FSS: (**a**) sensing element bonding, (**b**) encapsulation layer assembly, (**c**) force-concentrating layer assembly, (**d**) photograph of the FSS prototype.

Based on the prepared liquid metal pressure-sensing element with a variable cross-section, the FSS was integrated as follows:

(1) Sensing element bonding: As shown in Figure 6a, a flexible cubic core made of polydimethylsiloxane (PDMS) was fabricated by mold casting, and silicone sealant was applied to the six surfaces of the cubic core to bond the fabricated pressure-sensing elements with variable cross-sections. The assembly was then cured in a vacuum oven to ensure complete adhesion.

(2) Encapsulation layer assembly: The PDMS encapsulation layer was cured and peeled off the 3D-printed mold. As shown in Figure 6b, the cubic core with six attached sensing elements was assembled with two PDMS-encapsulation layers using a silicone sealant.

(3) Force-concentrating layer assembly: The force-concentrating layers were prepared using 3D-printed mold casting. Finally, the six force-concentrating layers were bonded to the outside using a silicone sealant, thereby completing the integration of the FSS, as shown in Figure 6c. A photograph of the FSS prototype is shown in Figure 6d, which has a symmetrical manufacturing structure.

## 5. Experimental Results

### 5.1. Performance of Variable Cross-Section Liquid Metal Pressure Sensing Elements

**Figure 7:**
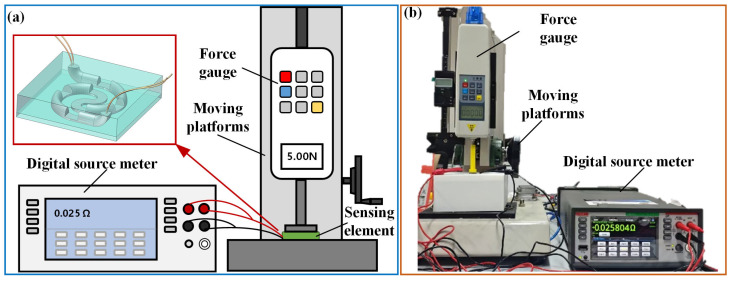
(**a**) Schematic and (**b**) photograph of the performance testing platform for the liquid metal pressure-sensing element.

To study the performance of the liquid metal pressure-sensing element with a variable cross-section, a laboratory-made performance testing platform was set up, as shown in Figure 7. A vertical tension testing machine (HPA, Handpi Instruments Co.,Ltd., Yueqing, China) with a force gauge was used to apply pressure to the sensing element. The digital source meter (Keithley 2450 SMU, Tektronix Inc., Beaverton, OR, USA) was set to the four-wire method resistance measurement mode and connected to the four electrodes of the pressure-sensing element.

**Figure 8:**
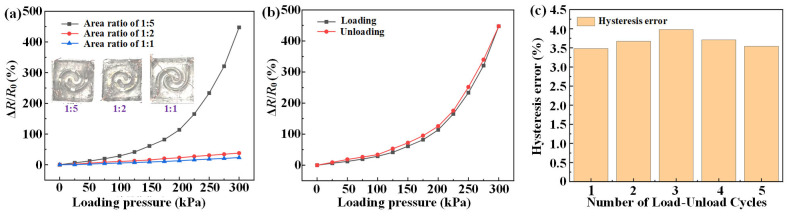

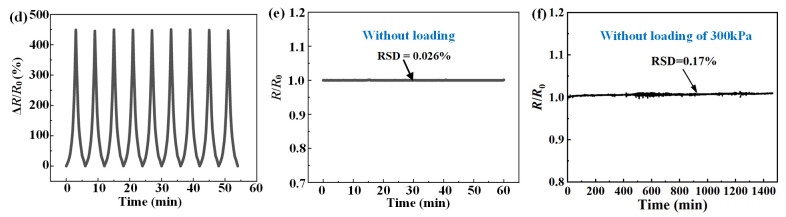
Performance of the variable cross-sectional liquid metal pressure-sensing element: (**a**) relative resistance change with stress, (**b**) hysteresis, (**c**) hysteresis error in five cycles, (**d**) repeatability and stability, (**e**) without loading, and (**f**) with loading of 300 kPa.

Sensitivity represents the resistance variation of the pressure-sensing element under the applied pressure, which can be calculated as follows [28].

(8) $$S=\frac{\Delta R/R_{0}}{\Delta P}$$

where Δ*R* and *R*_0_ are the resistance change and the initial value of the pressure-sensing element, respectively, and Δ*P* is the applied pressure change. To investigate the effect of the channel structure on sensitivity, variable cross-section channels with area ratios of 1:2 and 1:5, as well as a constant cross-section with an area ratio of 1:1, were fabricated. The dimensions of the three sensing elements were consistent, and both the length and overall cross-sectional areas were identical. The relative resistance changes of the different sensing elements under pressures ranging from 0 to 300 kPa are shown in Figure 8a. The sensing element with an area ratio of 1:5 exhibited the highest relative resistance change of approximately 450%. The sensing element with an area ratio of 1:2 increased slowly and linearly to approximately 50%. Therefore, the variable cross-section liquid metal pressure-sensing element with an area ratio of 1:5 shows the highest sensitivity of 1.5%kPa^−1^, whereas the sensitivity of the constant cross-section sensing element is 0.078% kPa^−1^. Therefore, the optimal area ratio of the liquid metal pressure-sensing element with a variable cross-section was 1:5.

Hysteresis represents the non-coincidence of the pressure sensing element output during the loading and unloading processes. It is usually quantified by the hysteresis error, which is calculated as [29]:

(9) $$\delta_{H}=\frac{\Delta H_{max}}{Y_{FS}}\times 100\%$$

where Δ*H_max_* represents the maximum output difference between the loading and unloading processes, and *Y_FS_* is the full-scale output of the sensing element. To quantitatively assess the impact of hysteresis, the sensor element was subjected to five identical tests. The average *δ_H_* of the sensor element during the five loading-unloading tests was approximately 3.5%, as illustrated in Figure 8b. The maximum *δ_H_* was 3.98%, indicating good hysteresis characteristics, as shown in Figure 8c. The relatively low hysteresis characteristics of the sensor element are due to the synergistic effect of the elastic PDMS and self-healing liquid metal under practical cyclic loading conditions.

To thoroughly study the repeatability of the variable cross-sectional liquid metal pressure-sensing element, nine repeated loading-unloading experiments were conducted within a range of 0–300 kPa. As shown in Figure 8d, the relative resistance variation of the sensing element during the nine repeated loading-unloading experiments exhibited highly overlapping curves, demonstrating a high degree of output consistency. Therefore, in the complex and varying internal stress environment of the SRM propellant, this sensing element can accurately measure stress variations.

The static stability of the sensing element was evaluated to ensure the reliability of the FSS embedded in the propellant. The sensing element was tested under both initial conditions and a loading of 300 kPa for 24 h. To quantify stability, the relative standard deviation (RSD) was employed, which could be calculated as follow

(10) $$RSD=\frac{\sqrt{\frac{\sum_{i=1}^{n}{\left(R_{i}\;-\;\bar{R}\right)}^{2}}{n\;-\;1}}}{\bar{R}}\times 100\%$$

where *R_i_* and $\bar{R}$ are the measured and average resistances under the same stress, respectively, and *n* is the number of the resistance data points collected in 24 h. As shown in Figure 8e,f, RSD at an under loading of 300 kPa was 0.17%. Moreover, the sensitivity and stability of the developed liquid metal sensing element were compared with those of other reported works [30,31,32,33]. As shown in Table 1, the liquid metal sensing element developed in this study achieved a relatively balanced sensitivity and stability. Therefore, the sensing element exhibits excellent performance under different stresses for the long-term health monitoring of propellants.

### 5.2. Simulated Propellant Experimental Validation of FSS

**Figure 9:**
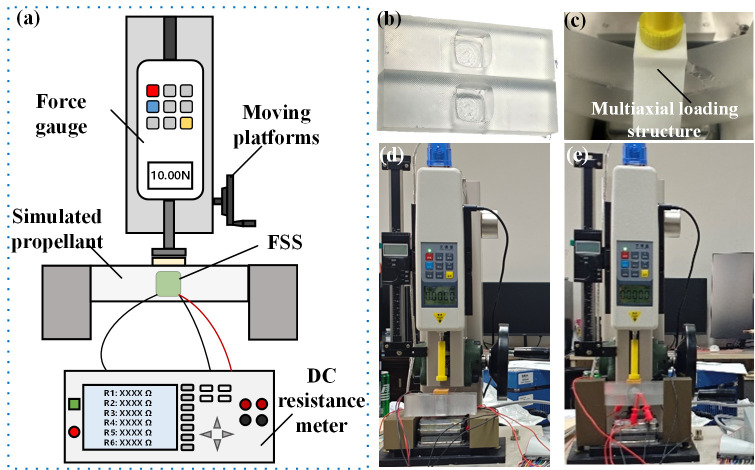
(**a**) Schematic of the experimental setup for the embedded FSS in the simulated propellant, photograph of (**b**) simulated propellant parts, (**c**) multiaxial loading structure, and the experimental setup under (**d**) uniaxial normal loading and (**e**) multiaxial loading.

To achieve cost efficiency and safety considerations, the proposed FSS prototype was verified in a simulated propellant environment, as shown in Figure 9a. The FSS was embedded in the simulated propellant and cured with a rubber paste. The simulated propellant was fixed using two fixtures and loaded using force gauges on a moving platform. The simulated propellant module comprised two parts, both featuring a structure with curved grooves that closely matched half of the sensor’s outer surface, as shown in Figure 9b. A DC resistance meter (TH2518A, Tonghui Electronic Co. Ltd., Changzhou, China) with a resistance accuracy of 0.05% was used to simultaneously measure the resistance of the six sensing elements in the FSS. Multiple resistance values can be rapidly obtained in a short time by polling. Based on the collected resistance data, the stress differences measured in each direction can be calculated. Using the multiaxial loading structure shown in Figure 9c, the FSS in the simulated propellant can be deformed under complex conditions. Photographs of the experimental setup for uniaxial normal and multiaxial loading are shown in Figure 9d,e, respectively.

**Figure 10:**
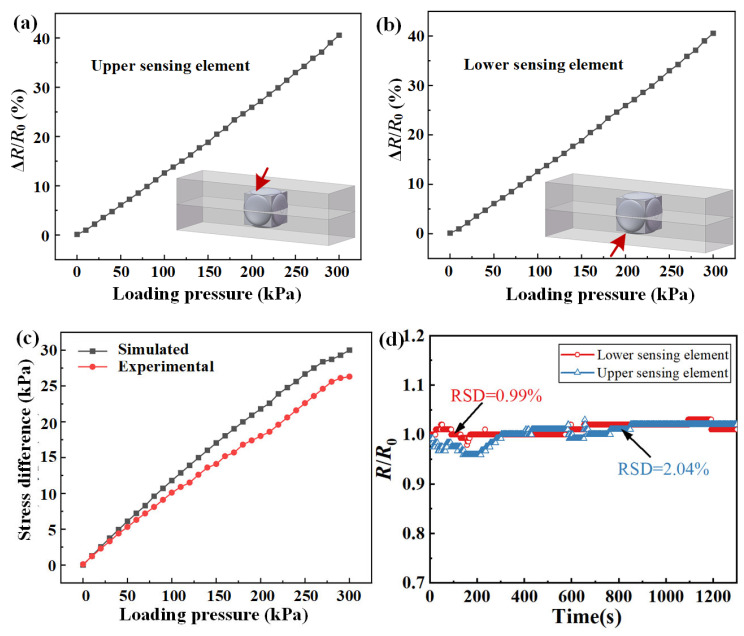
Experimental results of FSS embedded in simulated propellant under uniaxial loading: relative resistance change of (**a**) upper and (**b**) lower sensing elements, (**c**) comparison between simulated and experimental stress differences of FSS, (**d**) multiaxial loading stability under pressure of 200 kPa.

Using the constructed experimental platform, uniaxial normal pressures ranging from 0 to 300 kPa were applied to the simulated propellant. The measured relative resistance changes in the upper and lower sensing elements of the FSS are illustrated in Figure 10a,b, respectively. Based on the calibrated data from the coaxial sensing elements, a segmented interpolation method was employed to evaluate the measured stress data corresponding to each sensing element, as shown in Figure 10c. As the load increased, the difference between the experimental and simulated stresses increased. Under the full range of 300 kPa, the difference between the experimental and simulated stress is approximately 10 kPa; therefore, the overall trend remains largely consistent. To investigate the stability of the FSS embedded in the simulated propellant environment, a uniaxial normal pressure of about 200 kPa was applied for 20 min. As shown in Figure 10d, the maximum RSD under this condition was 2.04%.

**Figure 11:**
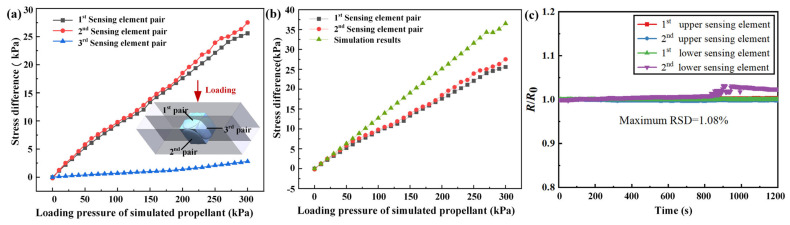
Experimental results of multiaxial loading of simulation propellant: (**a**) triaxial stress difference of FSS, (**b**) comparison between simulated and measured stress difference of FSS. (**c**) multiaxial loading stability under pressure of 90 kPa.

A multiaxial loading structure was used to apply a multidimensional pressure of 0–300 kPa to the simulated propellant. The triaxial stress differences measured by the three pairs of sensing elements are shown in Figure 11a. The first and second pairs of sensing elements were symmetrical and under identical loading conditions. The stress differences measured by these two pairs of sensing elements were highly consistent, with a maximum measurement difference of approximately 2 kPa. A slight stress difference ranging from 0–3 kPa was measured by the third pair owing to minor factors, such as the placement of the sensors. These results indicate that the FSS has good symmetry and consistency among pairs of sensing elements in different directions. A comparison between the experimental and simulated stress differences in the multiaxial loading test is shown in Figure 11b. It can be seen that the stress differences measured by the two symmetrical pairs of sensing elements match the simulation data very well in the 0–50 kPa range. However, after 50 kPa, as the load increased, there was approximately a 20% measurement deviation between the two pairs of sensing elements. Because the PDMS in the finite element modeling is simplified as elasticity, the deviation may be due to the viscoelastic behavior of PDMS and changes in the resistivity of the liquid metal in the sensing elements under these conditions. In addition, manufacturing and assembly errors may influence the output performance of the FSS. It is significantly difficult to completely eliminate the viscoelastic drift of the embedded FSS in SRM propellants. In future work, machine learning algorithms should be applied to assist with damage diagnostics. To investigate the stability of the FSS embedded in the simulated propellant environment, a multiaxial normal pressure of about 90 kPa was applied for 20 min. As shown in Figure 11c, the maximum RSD under this condition was 1.08%. Therefore, the developed FSS should feature excellent stability for practical applications in SRM propellant health monitoring.

## 6. Conclusions

To address the challenge of achieving in situ three-dimensional stress changes in SRM propellants, this study proposes a novel structural design for an FSS. A liquid metal pressure-sensing element with a variable cross-section was designed first, and finite element simulations were conducted to verify its advantages in improving sensitivity. A prototype of the pressure-sensing element was fabricated and experimentally characterized. The results show that the sensing element with an area ratio of 1:5 achieves a sensitivity coefficient of 1.5%kPa^−1^ at 300 kPa, with a maximum hysteresis error of 3.98% and stability error of 0.17% within 24 h. The FSS was then integrated and characterized on a simulated propellant experimental platform. By comparing with the simulation, the experimental results demonstrated the feasibility of using the FSS to detect internal stress changes in the SRM propellant. Future research will further explore the embedded integration method and application verification of this technology in actual SRM propellant.

## Figures and Tables

**Table 1:** Comparison of sensitivity and stability between this work and previously reported flexible sensors.

	Sensing Mechanism	Sensitivity	Stability Deviation
Ref. [30]	capacitive	0.42 kPa^−1^	1.57%
Ref. [31]	piezoresistive	0.705 kPa^−1^	0.7%
Ref. [32]	piezoresistive	0.061 mA kPa^−1^	5%
Ref. [33]	piezoresistive	-	5.2%
This work	piezoresistive	1.5% kPa^−1^	0.17%

## Data Availability

The original contributions presented in this study are included in the article/Supplementary Material. Further inquiries can be directed to the corresponding authors.

## Supplementary Materials

The following supporting information can be downloaded at: https://www.mdpi.com/article/10.3390/mi17010057/s1, Video S1: FSS loading stability.
